# Brain over muscle: central mechanisms predominate in gait impairment among older adults with type 2 diabetes mellitus

**DOI:** 10.3389/fnagi.2026.1787033

**Published:** 2026-06-17

**Authors:** Zheping Zhou, Zhaozhe Wang, Guiyu Li, Honghong Zhang, Zongyi Chen, Yexuan Xia, Yueju Wang, Ji Hu

**Affiliations:** 1 Department of Endocrinology and Metabolic Medicine, The Second Affiliated Hospital of Soochow University, Suzhou, China; 2 Department of Geratology, The First Affiliated Hospital of Soochow University, Suzhou, China; 3 Department of Geratology, Afiliated Changshu Hospital of Nantong University, Changshu, China; 4 Department of Radiology, The Second Affiliated Hospital of Soochow University, Suzhou, China

**Keywords:** central mechanisms, cognitive function, gait impairment, hierarchical regression, hippocampal atrophy, older adults, type 2 diabetes mellitus

## Abstract

**Background:**

Gait impairment is common in older adults with type 2 diabetes mellitus (T2DM). This complication increases risk of falls, disability, and mortality. Although gait disorders are often associated with peripheral deficits, emerging evidence suggests that central mechanisms may play an important role. The relative contributions of central and peripheral factors across gait parameters remain unclear.

**Methods:**

In this cross-sectional study, gait parameters were assessed using wearable inertial sensors in 41 older adults with T2DM and 55 age-matched controls. Central factors included medial temporal atrophy (MTA) on magnetic resonance imaging (MRI) and global cognition (MoCA/MMSE). Grip strength was used as the index of peripheral function. Hierarchical regression quantified the incremental variance explained by central versus peripheral factors after adjusting for demographic and vascular confounders.

**Results:**

Compared with the control group, the T2DM group showed significantly worse gait performance, including a reduction in gait speed of about 35% (*p* < 0.001). For gait speed, central factors (MTA and cognition) explained most of the model’s explained variance, whereas grip strength explained little and was not an independent predictor. Both central and peripheral factors contributed independently to cadence.

**Conclusion:**

Hippocampal atrophy and cognitive dysfunction are independently associated with slowed gait speed in the elderly with T2DM. Cadence reflects combined effects of central and peripheral influences. These findings suggest that future intervention research should explore a shift toward from traditional peripherally focused approaches to brain-centered strategies for maintaining motor ability in the future. These findings are cross-sectional and associative; causal inferences require longitudinal validation.

## 1 Introduction

According to the latest data, an estimated 589 million to 828 million people worldwide are living with type 2 diabetes mellitus (T2DM), and the number is projected to continue to grow in the coming decades (Kalyani et al., 2025). T2DM constitutes a critical public health crisis affecting the global population in modern society. The economic burden of T2DM among the elderly is quite heavy, particularly among Medicare beneficiaries aged over 65 years, in whom diabetes-related complications generate substantial healthcare costs. Therefore, the need for effective prevention and management strategies in this vulnerable population is very urgent (Wang et al., 2022). Although T2DM has long been recognized for its metabolic complications, its impact on body function, especially gait, should have received more attention than in current clinical practice and research.

Compared with their peers without diabetes, the older adults with T2DM showed significantly different gait alterations, with slower walking speed and reduced vertical and anteroposterior ground reaction forces (Katoulis et al., 1997). These gait changes are far more than subtle kinematic changes. They have a profound practical impact on the safety and quality of life of patients. T2DM individuals face an almost 1-3-fold increased risk of falling, and their gait impairment can also predict accelerated functional decline, premature erosion of self-care capacity, and a higher risk of death (Freire et al., 2024). Geriatric medicine regards gait speed as a “frailty vital sign”, which reflects its robust predictive value for overall health and future outcomes (Wildes, 2019). Understanding the potential mechanisms of gait impairment in the T2DM population is not only an academic issue, but also a critical step in developing targeted interventions for this rapidly growing population that can maintain mobility and prevent disability.

At present, the etiology of gait disorder in the T2DM population has not been fully clarified, but the existing research mainly focuses on the two primary mechanistic frameworks: central nervous system pathology and peripheral mechanisms. Growing evidence suggests T2DM promotes cognitive decline, especially in the domains of language/semantic categorization, attention/working memory and episodic memory. The integrity of these functions is crucial to the coordination of motor performance. In the elderly with T2DM, the decline of motor function, especially the deterioration of gait speed, can predict the accelerated cognitive decline, suggesting the shared neuropathological mechanisms (Ganmore et al., 2020). Longitudinal neuroimaging studies showed that T2DM patients demonstrated accelerated brain atrophy compared with the control population, showing greater lateral ventricular enlargement and increased white matter hyperintensity progression over a four-year follow-up (de Bresser et al., 2010). The hippocampus has long been regarded as a memory-related structure. But today, increasing research suggests that hippocampus plays a critical role in spatial navigation and motor coordination. The reduction in the volume of the hippocampus has been thought to be directly related to the slower walking speed of the T2DM elderly, and changes in brain structure may directly compromise locomotor control (Joshi et al., 2023).

Another line of mechanism research focuses on peripheral pathology. T2DM is well-established as a cause of peripheral complications. T2DM peripheral neuropathy weakens the sensory feedback from the feet, thus impairing proprioception and balance control (Brown et al., 2015). The muscle strength of the T2DM elderly declines more rapidly, and the loss of muscle mass and muscle quality accelerates; this trend persists after accounting for chronic inflammation, body composition and physical activity levels (Park et al., 2007). As a proven alternative indicator of overall muscle function, grip strength can independently predict the risk of fractures in the elderly of T2DM; for each 5 kg increase in grip strength, the risk of fractures is reduced by 18% (Dufour et al., 2021).

Most existing studies explore central factors and peripheral mechanisms separately, making it difficult to directly compare the relative importance of these two pathways. This knowledge gap is of great clinical significance. The development of effective intervention measures requires clarity about where the therapeutic efforts should be directed. Cognitive training and nerve protection strategies target the central pathway, while strength training and peripheral nerve management are aimed at peripheral mechanisms (Castellote-Caballero et al., 2024; Orlando et al., 2024). Both pathways are likely to contribute to gait disorders, but their relative importance remains unclear. If the central factors explain most of the gait variation and the contribution of peripheral factors is small, the intervention strategy should be fundamentally adjusted accordingly. The limited resources of the healthcare system and the limited financial capacity of patients make it necessary to determine the priority of intervention.

This study aims to address this gap through a comprehensive cross-sectional study. We systematically evaluated the central factors (cognitive function, hippocampal volume scores derived from MRI) and peripheral factors (with grip strength as an indicator of overall muscle function) in participants. The gait was evaluated under controlled conditions, and indicators such as walking speed, cadence and stride length were recorded. By integrating and analyzing the central factors and peripheral factors, we aimed to gain an in-depth understanding of the mechanisms of gait impairment in T2DM patients. These findings are expected to provide a basis for the development of more targeted and mechanism-specific interventions to maintain the mobility of older adults with T2DM.

## 2 Methods

### 2.1 Study design and participants

All participants were recruited from the Geriatric Clinic at the First Affiliated Hospital of Soochow University. The study protocol received approval from the Ethics Committee of both the First and Second Affiliated Hospitals of Soochow University. Written informed consent was obtained from all participants and their caregivers prior to enrollment.

#### 2.1.1 Inclusion criteria

Participants were required to meet the following criteria: (1) age ≥60 years; (2) ability to walk independently for at least 10 min without assistive devices; and (3) absence of severe systemic physical diseases.

#### 2.1.2 T2DM diagnosis

T2DM was diagnosed by an endocrinologist based on medical records according to American Diabetes Association (ADA) criteria, with independent verification by a second reviewer. For participants with T2DM, additional inclusion criteria included: (1) physician-confirmed T2DM diagnosis per ADA guidelines; (2) absence of acute hyperglycemic episodes (e.g., diabetic ketoacidosis, hyperosmolar hyperglycemic state) or severe hypoglycemia requiring medical intervention within the past 3 months; and (3) stable diabetes management with no modifications to oral hypoglycemic agents or insulin regimens within 3 months before assessment.

#### 2.1.3 Exclusion criteria

Participants were excluded if they met any of the following criteria: (1) Neuromuscular or musculoskeletal disorders potentially causing gait abnormalities (e.g., stroke, Parkinson’s disease, severe arthritis, significant lower limb trauma or surgery); (2) Cognitive or psychiatric conditions precluding cooperation with assessments, regardless of diagnostic classification; (3) Severe sensory impairments, including moderate-to-severe non-proliferative or proliferative diabetic retinopathy confirmed by ophthalmologic examination, or significant hearing impairment; (4) Cardiovascular, peripheral vascular, or respiratory conditions with functional limitations to ambulation, including symptomatic cardiovascular disease (myocardial infarction history, NYHA class III–IV heart failure, unstable angina, or clinically significant arrhythmias); (5) Recent medication use (within 14 days prior to assessment) with known effects on gait or cognition, including benzodiazepines, antipsychotics, tricyclic antidepressants, or other strongly anticholinergic agents, opioid analgesics, skeletal muscle relaxants, and sedative-hypnotics; (6) Severe diabetic complications independently impairing gait performance, such as active diabetic foot ulcers, or Charcot neuroarthropathy; (7) Acute conditions likely to alter gait stability or speed, including active infections, acute vertigo, severe pain flares, symptomatic orthostatic hypotension, unplanned hospitalization or major treatment changes within 2 weeks, or documented falls within 3 months resulting in persistent gait alterations.

Following enrollment, all participants underwent comprehensive assessment including medical history collection, neuropsychological testing, and evaluation of activities of daily living.

### 2.2 Sociodemographic characteristics and cognitive assessment

Sociodemographic data were collected through structured face-to-face interviews, including age, gender, height, weight, years of education, and a medical history of hypertension.

#### 2.2.1 Cognitive assessment

Global cognitive function was evaluated using two validated instruments: the Mini-Mental State Examination (MMSE) and the Montreal Cognitive Assessment (MoCA; Beijing version), with one point added for participants with < 12 years of education to adjust for educational attainment (Jia et al., 2021).

#### 2.2.2 Functional assessment

The Instrumental Activities of Daily Living Scale (IADL) was administered to assess participants’ capacity for independent living and performance of complex daily activities (Ding et al., 2024). Grip strength was assessed as the average of two measurements of the dominant hand, selected as a validated proxy for overall muscle health and functional decline in older adults.

### 2.3 Gait assessment

Spatiotemporal gait parameters were assessed using the wireless APDM Movement Monitoring inertial sensor system (APDM Inc., Portland, OR, United States) (Beichert et al., 2024). Six Opal sensors were positioned on participants’ bodies: one on the lower back, two on the shanks, one on the sternum, and two on the wrists. Gait features were automatically analyzed using APDM’s Mobility Lab™ software.

Participants were asked to ambulate at a self-selected, comfortable speed while wearing their everyday footwear. They repeatedly traversed a 7-m straight walkway for 2 min, performing a 180° turn at each end of the walkway (endpoints marked with tape). Prior to testing, standardized verbal instructions and demonstrations were provided to all participants. An experienced researcher walked behind each participant as a safety precaution to prevent falls. Outcome measures of interest were extracted.

### 2.4 Magnetic resonance imaging (MRI) data acquisition

All cerebral MRI scans were performed on a GE Signa HDxt 3.0 T scanner (Signa HDxt, GE Healthcare, Milwaukee, WI, United States) by a single experienced physician to ensure consistency.

Imaging parameters:

- 3D-T1-weighted imaging (3D-T1WI): TR = 6.52 ms, TE = 2.80 ms, flip angle = 12°, slice thickness/gap = 1.0/0 mm, field of view (FOV) = 260 × 260 mm, voxel size = 1 × 1 × 1 mm^3^, acquisition time = 4.25 min.
- T2-FLAIR-weighted imaging: TR = 8,000 ms, TE = 147 ms, TI = 1,500 ms, slice thickness/gap = 5.0/1.5 mm, FOV = 240 × 240 mm, matrix = 256 × 192.

Image analysis: Two experienced radiologists independently evaluated baseline images for white matter hyperintensities (WMH) and hippocampal atrophy using validated visual rating scales, blinded to all clinical information. Disagreements were resolved through consensus discussion. WMH severity was quantified using the Fazekas scale, while the medial temporal atrophy (MTA) rating scale was employed to assess bilateral hippocampal atrophy. Visual MTA scoring was selected for clinical feasibility and epidemiological comparability, with inter-rater reliability confirmed 𝜅 = 0.82, though automated volumetry would offer greater sensitivity. MTA scores from both hemispheres were recorded separately.

### 2.5 Statistical analysis

All statistical analyses were performed using R software (version 4.5.1). Normality of continuous variables was assessed using Shapiro–Wilk tests. Data are presented as mean ± standard deviation (SD) for normally distributed variables, median [interquartile range] for non-normally distributed variables, or frequency (percentage) for categorical variables. Post-hoc power analysis was conducted to evaluate the statistical power for detecting between-group differences.

Independent samples *t*-tests were used for normally distributed continuous variables, Mann–Whitney U tests for non-normally distributed variables, and chi-square tests for categorical variables. Effect sizes were calculated as Cohen’s d for *t*-tests and rank-biserial correlation (r) for Mann–Whitney *U* tests, with values interpreted as small (*d* = 0.2, *r* = 0.1), medium (d = 0.5, *r* = 0.3), or large (*d* = 0.8, *r* = 0.5). Spearman correlation coefficients were computed within the T2DM group to examine relationships between gait parameters and hypothesized mechanistic factors.

To quantify independent and incremental contributions of central vs. peripheral factors, hierarchical multiple regression models were fit in the T2DM group: Model 1 adjusted for age, sex, education, and hypertension; Model 2 added MTA and MoCA; Model 3 added grip strength. We reported R^2^, adjusted *R*^2^, standardized *β* and blockwise Δ*R*^2^ with F-change tests. Multicollinearity was evaluated using variance inflation factors (VIF < 5). Sensitivity analyses included adding interaction terms and bootstrap validation (1,000 resamples) for Δ*R*^2^ with percentile 95% confidence intervals (CI).

## 3 Results

### 3.1 Participant characteristics

A total of 96 participants were enrolled in this study, comprising 55 individuals in the control group and 41 in the T2DM group. Baseline characteristics and between-group comparisons are presented in Table 1.

**Table 1:** Participant characteristics and group comparisons between control and T2DM groups.

Category	Variable	Control	T2DM	*p* value
Sample size	*n*	55	41	
Demographics	Age (years)	73.36 ± 7.49	74.83 ± 7.79	0.356
	Gender, female *n* (%)	23 (41.8%)	22 (53.7%)	0.346
	Education (years)	11 [8, 14]	8 [5, 11]	0.120
	BMI (kg/m^2^)	22.79 ± 3.46	22.62 ± 2.95	0.796
	Hypertension, yes *n* (%)	20 (36.4%)	23 (56.1%)	0.086
Cognitive and functional status	MMSE	27 [25, 28]	23 [14, 27]	0.002**
	MoCA	24 [20.5, 26]	19 [11, 24]	0.001**
	IADL	12 [12, 13]	13 [12, 16]	0.005**
Muscle strength	Grip strength (kg)	24.5 [20.2, 29.5]	18.1 [10.6, 26.1]	0.004**
Neuroimaging	Fazekas score	2 [1, 3]	2 [1, 3]	0.251
	MTA score (mean)	0.5 [0, 1]	1 [0, 2]	0.004**

Data are presented as mean ± SD for normally distributed continuous variables, median [Q1, Q3] for non-normally distributed continuous variables, or *n* (%) for categorical variables.

*p < 0.05, **p < 0.01, ***p < 0.001.

BMI, body mass index; MMSE, mini-mental state examination; MoCA, montreal cognitive assessment; IADL, instrumental activities of daily living; MTA, medial temporal atrophy; GCT, gait cycle time.

#### 3.1.1 Demographic characteristics

The two groups were well-matched for age (control: 73.36 ± 7.49 vs. T2DM: 74.83 ± 7.79 years, *p* = 0.356), gender distribution (female: 41.8% vs. 53.7%, *p* = 0.346), education years (11 [8, 14] vs. 8 [5, 11] years, *p* = 0.120), and body mass index (22.79 ± 3.46 vs. 22.62 ± 2.95 kg/m^2^, *p* = 0.796). Although the T2DM group exhibited a numerically higher prevalence of hypertension (56.1% vs. 36.4%), statistical significance was not achieved for this difference (*p* = 0.086).

#### 3.1.2 Cognitive and functional status

Participants with diabetes demonstrated significant cognitive impairment compared to controls. MMSE scores were lower in the T2DM group (23 [14, 27] vs. 27 [25, 28], *p* = 0.002), as were MoCA scores (19 [11, 24] vs. 24 [20.5, 26], *p* = 0.001), both showing moderate effect sizes. Furthermore, the T2DM group had significantly elevated IADL scores (13 [12, 16] vs. 12 [12, 13], *p* = 0.005), indicating greater dependency in instrumental activities of daily living.

#### 3.1.3 Muscle strength and neuroimaging

Grip strength was significantly reduced in the T2DM group (18.1 [10.6, 26.1] vs. 24.5 [20.2, 29.5] kg, *p* = 0.004), suggesting peripheral muscle weakness. Neuroimaging assessment revealed no significant between-group difference in Fazekas scores for white matter hyperintensities (2 [1, 3] vs. 2 [1, 3], *p* = 0.251). However, the T2DM group exhibited significantly higher MTA scores (1 [0, 2] vs. 0.5 [0, 1], *p* = 0.004), reflecting more severe medial temporal lobe atrophy.

### 3.2 Gait parameters

The T2DM group exhibited profound gait impairment across all major spatiotemporal parameters, with large effect sizes (Table 2). Gait speed was markedly reduced in the T2DM group (51.1 ± 15.36 vs. 78.28 ± 16.52 cm/s, *p* < 0.001, Cohen’s *d* = 1.695), representing approximately a 35% decrease. This deterioration was driven by both reduced stride length (70.01 ± 18.01 vs. 91.14 ± 18.13 cm, *p* < 0.001, *d* = 1.168) and decreased cadence (88.4 ± 13.81 vs. 103.13 ± 9.91 steps/min, *p* < 0.001, *d* = 1.256).

**Table 2:** Gait characteristics between control and T2DM groups.

Category	Variable	Control	T2DM	*p* value	Effect size
Sample size	*n*	55	41		
Gait parameters	Gait speed (cm/s)	78.28 ± 16.52	51.1 ± 15.36	<0.001	*d* = 1.695
	Cadence (steps/min)	103.13 ± 9.91	88.4 ± 13.81	<0.001	*d* = 1.256
	Stride length (cm)	91.14 ± 18.13	70.01 ± 18.01	<0.001	*d* = 1.168
	Gait cycle duration (s)	1.17 [1.09, 1.24]	1.38 [1.25, 1.5]	<0.001	*r* = 0.615
	Lateral step variability (cm)	6.88 [4.44, 9.39]	5.22 [4.79, 8.26]	0.094	*r* = −0.201
	Stance phase (%)	62.12 [60.51, 63.3]	64.06 [63.21, 65.33]	<0.001	*r* = 0.624
	Swing phase (%)	37.88 [36.7, 39.49]	35.94 [34.67, 36.94]	<0.001	*r* = −0.606
Turn parameters	Turn angle (degrees)	175.49 [163.43, 179.88]	151.26 [129.72, 166.52]	<0.001	*r* = −0.556
	Steps in turn	4 [3.67, 4.43]	4 [3.29, 5]	0.862	*r* = −0.021
	Turn duration (s)	2.49 ± 0.33	2.65 ± 0.46	0.060	*d* = −0.415
	Turn velocity (degrees/s)	156.34 ± 35.55	118.51 ± 29.32	<0.001	*d* = 1.145

Effect sizes are presented as Cohen’s d for *t*-test, rank-biserial correlation (*r*) for Mann–Whitney *U* test.

Gait cycle analysis revealed prolonged gait cycle duration in the T2DM group (1.38 [1.25, 1.50] vs. 1.17 [1.09, 1.24] s, *p* < 0.001, *r* = 0.615), accompanied by increased stance phase percentage (64.06% [63.21, 65.33%] vs. 62.12% [60.51, 63.30%], *p* < 0.001, *r* = 0.624) and correspondingly decreased swing phase percentage (35.94% [34.67, 36.94%] vs. 37.88% [36.70, 39.49%], *p* < 0.001, *r* = −0.606).

During turning tasks, the T2DM group demonstrated reduced turn angle (151.26° [129.72°, 166.52°] vs. 175.49° [163.43°, 179.88°], *p* < 0.001, *r* = −0.556) and 24% lower turn velocity (118.51 ± 29.32 vs. 156.34 ± 35.55 °/s, *p* < 0.001, *d* = 1.145). No significant difference was observed in lateral step variability between groups (*p* = 0.094).

**Figure 1:**
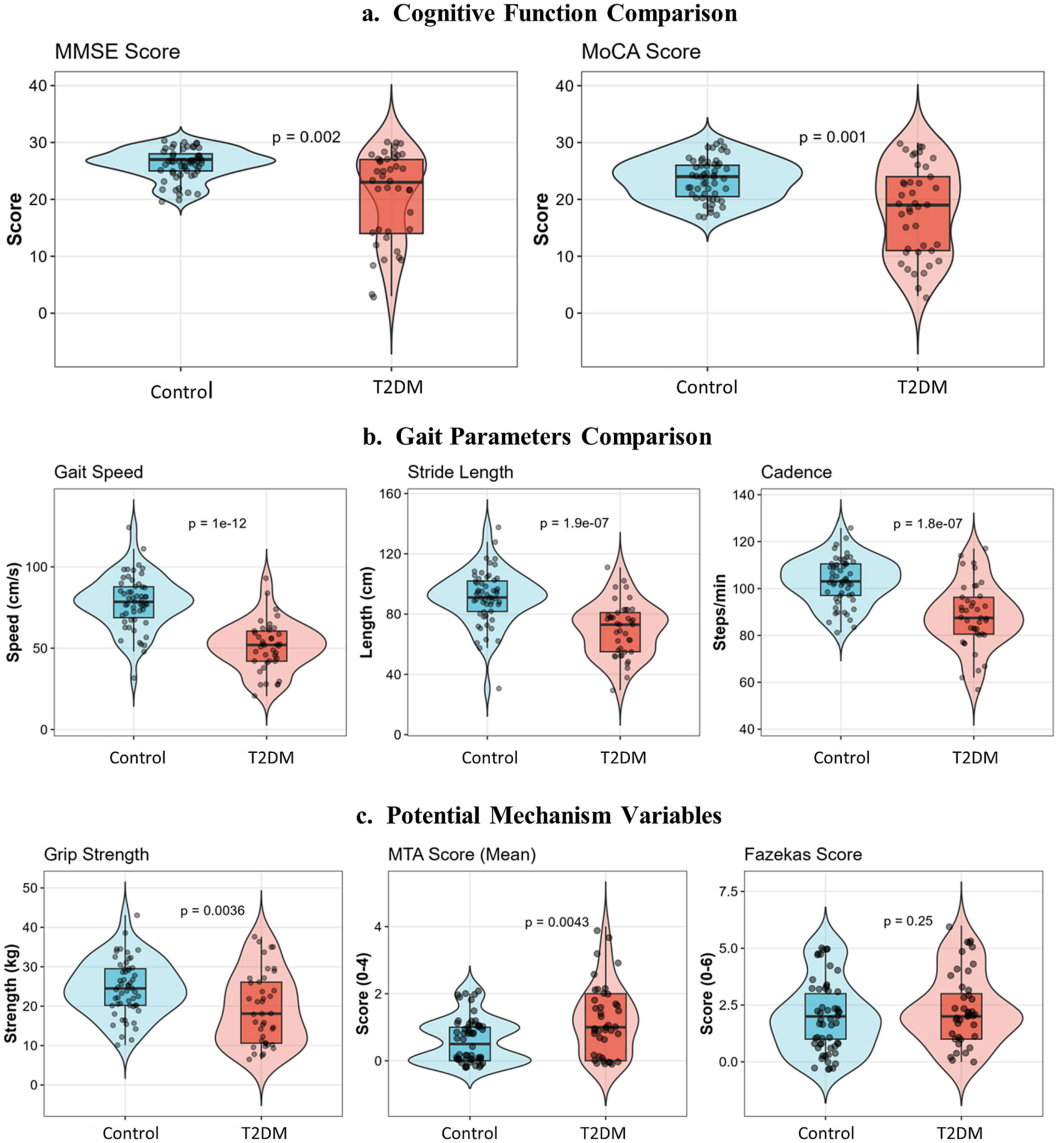
Between‑group differences in key clinical and gait variables. Violin plots comparing control vs. T2DM groups across **(a)** cognitive scores (MMSE, MoCA), **(b)** gait parameters (gait speed, stride length, cadence), and **(c)** potential mechanism variables (grip strength, MTA score, Fazekas score).

Between-group differences in key variables are visualized in Figure 1.

### 3.3 Correlation analysis

**Figure 2:**
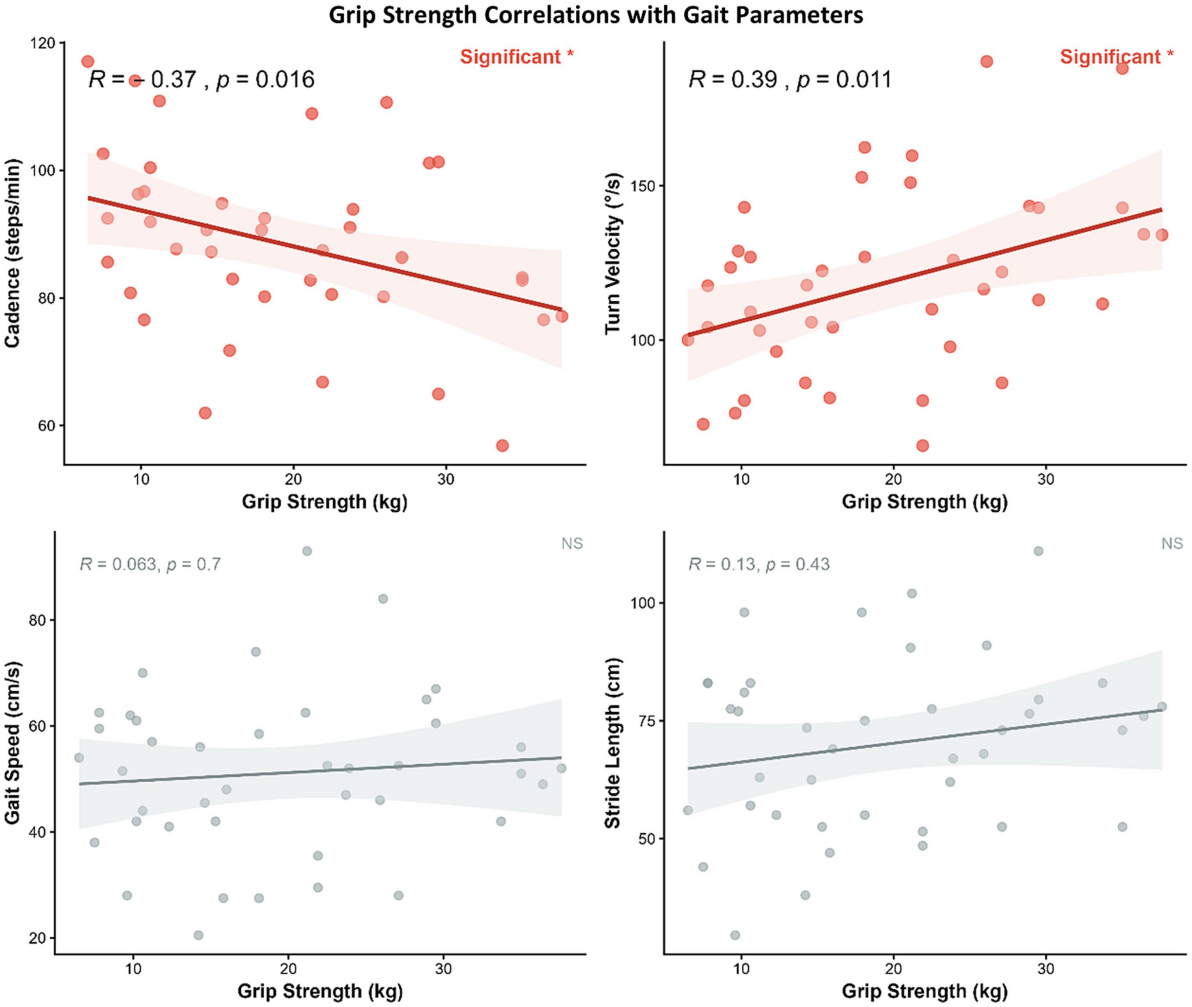
Correlation matrix between mechanistic factors and gait parameters. Correlation plot showing correlation coefficients between grip strength and gait parameters (gait speed, cadence, stride length, turn velocity).

**Figure 3:**
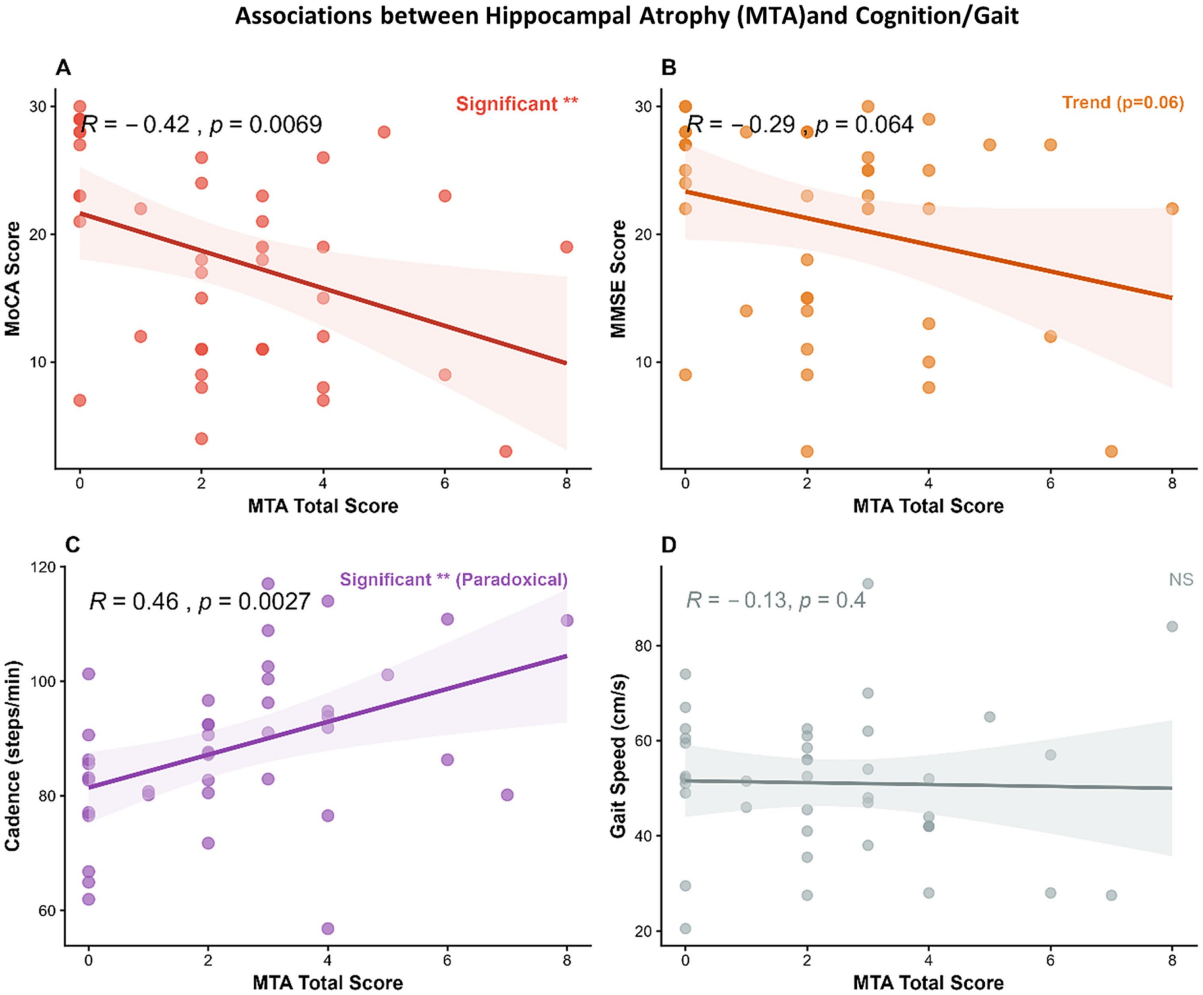
Correlation matrix between mechanistic factors and gait parameters. Correlation plot showing correlation coefficients between MTA scores, cognitive scores (MoCA, MMSE), and gait parameters (cadence, gait speed). Panels: **(A)** MTA vs. MoCA, **(B)** MTA vs. MMSE, **(C)** MTA vs. cadence, **(D)** MTA vs. gait speed.

Correlation analyses explored relationships between grip strength, hippocampal MTA grading, cognitive scores, and gait parameters (Figures 2, 3).

Grip strength correlations: Grip strength was significantly and inversely associated with cadence (*r* = −0.37, *p* = 0.016). Additionally, grip strength was positively correlated with turn velocity (*r* = 0.39, *p* = 0.011). However, correlations between grip strength and gait speed (*r* = 0.063, *p* = 0.700) or stride length (*r* = 0.13, *p* = 0.430) were not significant.

MTA and cognitive function correlations: MTA total score demonstrated a significant negative correlation with MoCA scores (*r* = −0.42, *p* = 0.007). A similar trend was observed for MMSE scores (*r* = −0.29, *p* = 0.064), although this difference did not reach statistical significance. In terms of gait parameters, cadence was significantly positively correlated with MTA total score (*r* = 0.46, *p* = 0.003). Gait speed showed no significant correlation with MTA (*r* = −0.13, *p* = 0.400).

### 3.4 Hierarchical multiple regression analysis

**Figure 4:**
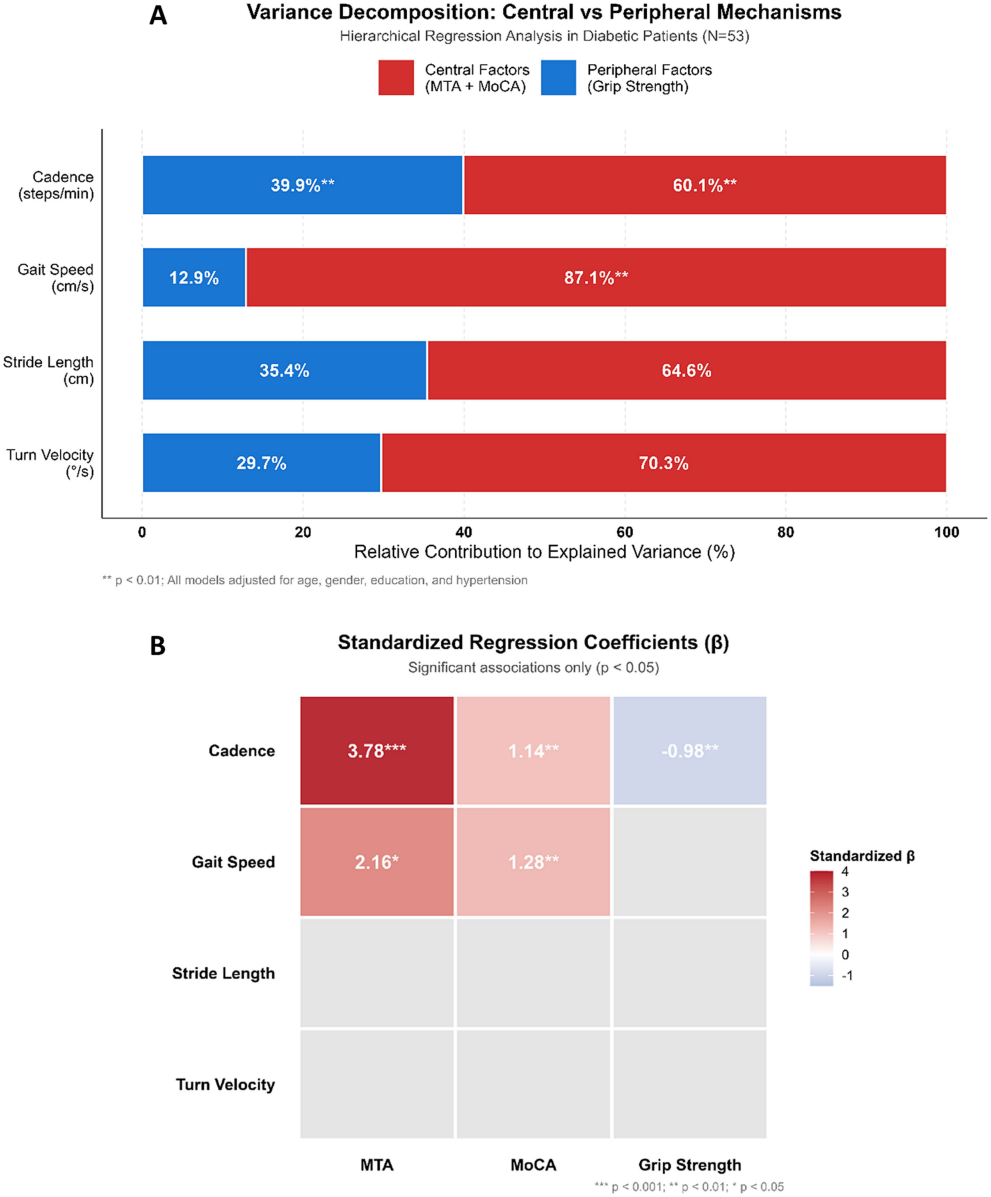
Hierarchical regression model results: variance decomposition and standardized coefficients. Panel **A**: Stacked bar chart illustrating the relative contributions (%) of central factors (MTA, MoCA) and peripheral factors (grip strength) to explained variance (ΔR^2^) for each gait parameter. Panel **B**: Heatmap of standardized regression coefficients (*β*) for significant predictors (*p* < 0.05).

**Figure 5:**
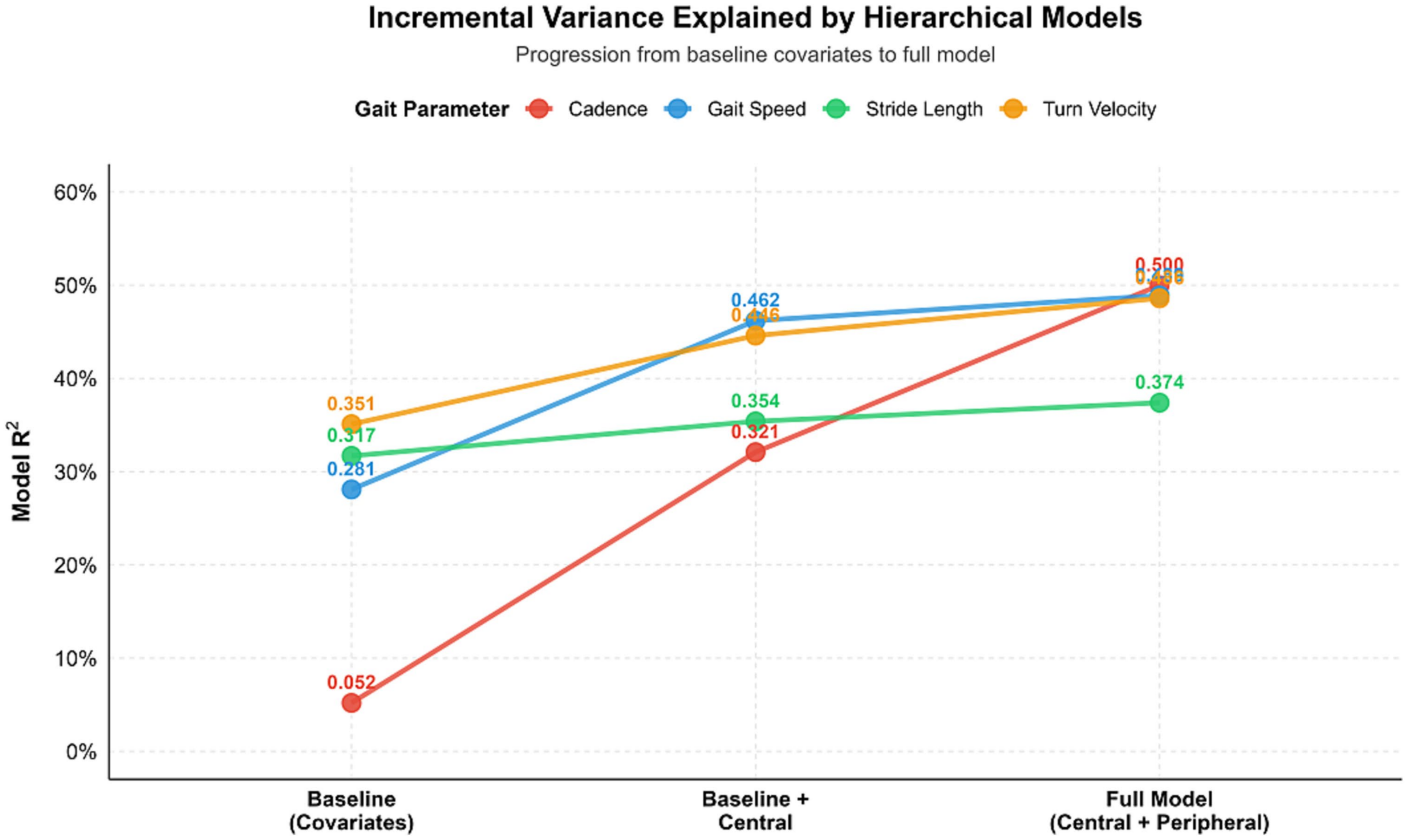
Line plot showing incremental *R*^2^ progression across Model 1 (baseline), Model 2 (+central factors), and Model 3 (+peripheral factors).

To disentangle the independent and incremental contributions of central (hippocampal atrophy, cognitive function) and peripheral (muscle strength) factors to gait parameters, we conducted three-step hierarchical multiple regression analyses (Table 3; Figures 4, 5).

**Table 3:** Hierarchical multiple regression analysis: independent and incremental contributions of central and peripheral factors to gait parameters.

Gait parameter	Central factors				Peripheral factors			Standardized coefficients		
	*R*^2^ (adjusted *R*^2^)	ΔR^2^	*p*	Contribution	ΔR^2^	*p*	Contribution	*β* MTA (*p*)	*β* MoCA (*p*)	*β* Grip (*p*)
Cadence (steps/min)	0.500 (0.394)	0.269	0.003**	60.1%	0.179	0.002**	39.9%	3.776 (0.000***)	1.143 (0.001**)	−0.984 (0.002**)
Gait speed (cm/s)	0.489 (0.381)	0.181	0.007**	87.1%	0.027	0.198	12.9%	2.159 (0.044*)	1.285 (0.001**)	−0.424 (0.198)
Stride length (cm)	0.374 (0.241)	0.037	0.391	64.6%	0.020	0.310	35.4%	0.348 (0.797)	0.794 (0.103)	−0.431 (0.310)
Turn velocity (°/s)	0.486 (0.377)	0.095	0.068	70.3%	0.040	0.118	29.7%	3.547 (0.082)	0.944 (0.186)	0.990 (0.118)

All models adjusted for age, gender, education years, and hypertension. ΔR^2^ = incremental *R*^2^ from adding factor block; Contribution = relative percentage of ΔR^2^ to total explained variance. ***p < 0.001; **p < 0.01; *p < 0.05.

Contribution (%) represents the proportion of the total incremental *R*^2^ (sum of ΔR^2^ for central and peripheral blocks) attributable to each block. It does not indicate the percentage of total variance in the gait parameter. A restricted sensitivity analysis (EPV ≈ 13.7:1) is reported in Supplementary Table S1.

#### 3.4.1 Cadence (steps/min)

Central factors contributed significantly to cadence variance (Δ*R*^2^ = 0.269, *p* = 0.003), accounting for 60.1% of the total explained variance. Peripheral factors also demonstrated a significant independent contribution (Δ*R*^2^ = 0.179, *p* = 0.002), representing 39.9% of explained variance. The final model explained 50.0% of cadence variance (adjusted *R*^2^ = 0.394). Standardized coefficients revealed that MTA score (*β* = 3.776, *p* < 0.001) and MoCA score (*β* = 1.143, *p* = 0.001) were positively associated with cadence, while grip strength showed a negative association (*β* = −0.984, *p* = 0.002).

#### 3.4.2 Gait speed (cm/s)

Central factors accounted for the majority of gait speed variance (Δ*R*^2^ = 0.181, *p* = 0.007), representing 87.1% of total explained variance, representing 87.1% of the variance explained by the hierarchical model blocks (i.e., Δ*R*^2^_central/total Δ*R*^2^ from all blocks). In contrast, peripheral factors contributed minimally and non-significantly (Δ*R*^2^ = 0.027, *p* = 0.198), accounting for only 12.9%. The final model explained 48.9% of variance (adjusted *R*^2^ = 0.381). MTA score (*β* = 2.159, *p* = 0.044) and MoCA score (*β* = 1.285, *p* = 0.001) were significant positive predictors, while grip strength was not (*β* = −0.424, *p* = 0.198).

#### 3.4.3 Stride length and turn velocity

Neither central nor peripheral factors reached statistical significance for stride length (ΔR^2^_central = 0.037, *p* = 0.391; ΔR^2^_peripheral = 0.020, *p* = 0.310) or turn velocity (ΔR^2^_central = 0.095, *p* = 0.068; ΔR^2^_peripheral = 0.040, *p* = 0.118).

#### 3.4.4 Multicollinearity assessment

All VIFs were below 2.5, indicating no problematic multicollinearity.

To address the modest events-per-variable ratio in the full model (EPV ≈ 5.9:1, *n* = 41), we conducted a restricted sensitivity analysis retaining only age, MTA, and MoCA (EPV ≈ 13.7:1). The central contribution to cadence remained robust (ΔR^2^ = 0.248, 90.4% of model *R*^2^, *p* = 0.004), and bootstrap 95% CIs excluded zero for all outcomes (Supplementary Table S1). For gait speed, the central contribution was attenuated (ΔR^2^ = 0.129, 54.1%, *p* = 0.055), yet remained the largest single explanatory block and was supported by bootstrap validation (95% CI 0.020–0.404). These findings suggest that central predominance is stable across model specifications, although the magnitude depends on covariate adjustment.

### 3.5 Sensitivity analyses

**Figure 6:**
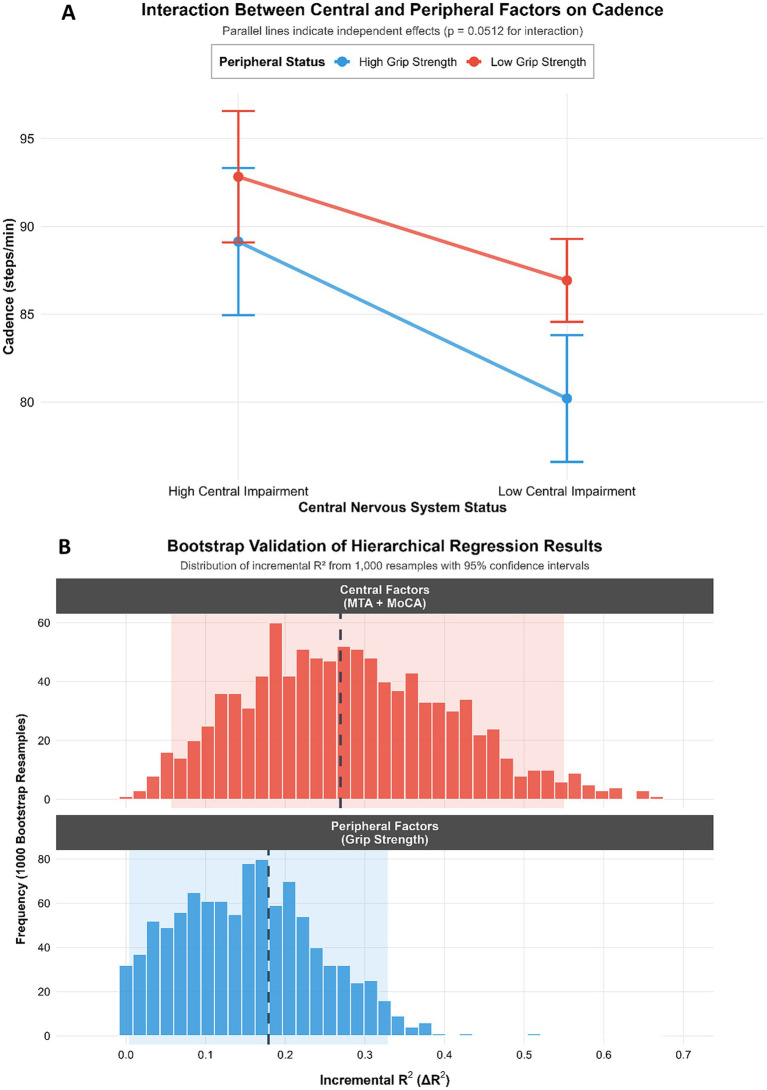
Sensitivity analyses: interaction testing and bootstrap validation. Panel **A** (Interaction Plot): Stratified line plot showing cadence as a function of central impairment status (high/low MTA × MoCA), stratified by grip strength level (high/low). Parallel lines indicate independent additive effects without interaction. Panel **B** (Bootstrap Validation): Histogram plot showing bootstrap distributions of ΔR^2^_central and ΔR^2^_peripheral with 95% CI (1,000 iterations). CI excluding zero confirm robust effects.

Interaction testing: The three-way interaction term (MTA × MoCA × grip strength) was not statistically significant (*p* = 0.051), indicating that central and peripheral factors contribute independently to cadence variance without synergistic interaction. Stratified analysis (Figure 6A) showed approximately parallel lines when cadence was plotted against central impairment status stratified by grip strength level, further supporting independent additive effects.

Bootstrap validation: Bootstrap resampling with 1,000 iterations confirmed the robustness of our hierarchical regression findings (Figure 6B). Across all bootstrap samples, both central and peripheral contributions remained statistically significant, with 95% CI excluding zero. Specifically, for cadence, the bootstrap 95% CI for ΔR^2^_central was [0.089, 0.462] and for ΔR^2^_peripheral was [0.053, 0.318], both excluding zero and confirming stable effect estimates.

## 4 Discussion

This study quantified the relative contributions of central and peripheral factors to the gait impairment of T2DM elderly patients. Our results showed that different gait parameters had distinct mechanistic profiles. Central factors were dominant for gait speed, accounting for 87% of the explained variance. Gait speed is arguably the most clinically meaningful gait metric. For cadence, we observed a mixed pattern: the central factors still dominated (60%), but peripheral factors also provided meaningful independent contributions (40%). However, neither set of factors significantly explained the variance in stride length or turning velocity, suggesting that there may be other mechanisms involved. These results challenged some existing assumptions about the gait impairment of T2DM people. Our research provided important insights into both theory and practice.

### 4.1 Central mechanisms dominate gait speed impairment

Central factors accounted for 87% of the variance that was explained by the hierarchical regression models (i.e., the incremental *R*^2^ from central factors divided by the sum of ΔR^2^ from central and peripheral blocks). This does not imply that central factors explain 87% of the total variability in gait speed; the final model explained 48.9% of total gait speed variance (adjusted *R*^2^ = 0.381). Both the MTA and MoCA scores were significant independent predictors. Therefore, we could think that the gait slowing of T2DM is not primarily associated with peripheral factors such as decreased muscle strength or neuropathy. When we added grip strength to the model, its incremental explained variance was negligible (ΔR^2^ = 0.027, *p* = 0.198). This pattern does not mean that muscle strength is irrelevant to gait; on the contrary, it suggests that in the context of T2DM-related cognitive and hippocampal structural changes, peripheral muscle strength is secondary in determining the walking speed.

The integrity of the hippocampus is associated with gait speed maintenance. The hippocampus is traditionally regarded as a memory structure, but new evidence shows that the hippocampus is a shared neural substrate for gait slowing and cognitive impairment, and it mediates the longitudinal association between the two in the elderly population (Rosso et al., 2017). Hippocampal place cells encode spatial location and provide causal support for navigation based on cognitive mapping (Robinson et al., 2020). In individuals with T2DM, hippocampal volume decreases due to chronic hyperglycemia, inflammation and vascular damage, and is associated with a decrease in the efficiency of the integration of spatial coding and motor planning, which coincides with slower gait (Hirabayashi et al., 2016). The consequences are not limited to episodic memory impairment, but also include the deterioration of individual walking function.

Compared with the MMSE, MoCA is more sensitive to executive functions, processing speed and memory. These domains are preferentially affected in T2DM through structural damage in specific hippocampal subregions (Vincent and Hall, 2015; Wu et al., 2025). The executive function determines the individual’s ability to coordinate complex multi-step behaviors. When the executive function declines, individuals appear to adopt a slower walking speed as a compensatory strategy to reduce the cognitive load and maintain safety (Morrison et al., 2024).

We did not observe significant differences between the T2DM group and the control group in vascular burden (Fazekas score). Given the well-confirmed association between T2DM and cerebrovascular disease, this result was somewhat unexpected (Georgakis et al., 2021). The reason may be that we have excluded individuals with a history of stroke and severe cognitive impairment, which may inadvertently exclude the individuals with the heaviest vascular burden. What is more noteworthy is that this “no difference” result suggests that in our T2DM sample, hippocampal atrophy may reflect a neurodegenerative process beyond the vascular mechanisms. T2DM can damage the hippocampus through multiple pathways independent of large-vessel or white matter vascular disease, including oxidative damage to insulin signaling proteins and subsequent AMPK dysregulation underlying neurodegeneration (Barone et al., 2021). Even in the absence of concurrent differences in vascular burden, hippocampal atrophy can still predict the walking speed, which further strengthens the view that the “hippocampal-specific neurodegeneration process” is the key correlate of T2DM gait impairment.

Recent studies have shown that the decline in executive function can predict the gait impairments of T2DM elderly patients over time, and cognitive resources become a limiting factor in this situation (Nóbrega-Sousa et al., 2020). In a four-year longitudinal follow-up, Sunghee Lee and colleagues found that changes in the volume of multiple brain regions (including hippocampus) were significantly associated with a decrease in the walking speed of the community-dwelling adults (Lee et al., 2019). The 87% central dominance we observed is particularly striking, suggesting that interventions targeting brain health may be more helpful in preserving gait speed than traditional approaches that focus solely on strength training or neuropathy management.

### 4.2 Cadence reflects mixed mechanisms: both central and peripheral contributions

The cadence showed significantly different mechanistic characteristics from the gait speed. Both central and peripheral factors provide significant independent contributions, with the central factors accounting for 60% and the peripheral factors accounting for 40% of the explained variation. Gait speed can be modulated by adjusting the step frequency or stride length, but cadence more specifically reflects the temporal rhythm of stepping. This rhythm depends on both the ability of motor planning and the mechanical ability to execute rapid lower limb movements.

In the multivariable models, cadence was independently and positively associated with both MTA score and MoCA score, while the grip strength is negatively correlated with cadence. The positive associations with MTA and MoCA seem counterintuitive at first. Some studies suggest that worse brain health will reduce cadence (Tian et al., 2019).

We acknowledge that the observed positive association between MTA and cadence is counterintuitive and requires cautious interpretation. Alternative explanations should be considered. First, higher MTA scores may reflect non-hippocampal pathology (e.g., global atrophy or comorbid neurodegenerative disease) that affects motor planning differently than focal hippocampal lesions (Xu et al., 2019). Second, the MTA scale is a coarse ordinal measure; participants with mild MTA may still have preserved compensatory capacity, whereas those with severe MTA might show cadence reduction—an effect masked by the linear modeling assumption (Wittens et al., 2024). Third, unmeasured confounders such as fear of falling or pain could drive both increased cadence and apparent structural changes (van der Kruk et al., 2022). Prior literature on MTA and gait is mixed: some studies report reduced cadence with worse MTA (Tian et al., 2019), while others find no association or paradoxical increases in step frequency among frail older adults (Xie et al., 2019). Our interpretation as a compensatory strategy is therefore plausible but speculative; longitudinal studies with volumetric hippocampal segmentation and detailed biomechanical assessment are needed to confirm this mechanism.

When individuals with more obvious hippocampal atrophy and more significant cognitive decline cannot maintain adequate stride length, they appear to compensate by adopting a more frequent and shorter pace pattern (Egerton et al., 2011; Xie et al., 2019). This is an adaptive strategy that allows individuals to maintain their mobility without the same level of motor control and balance that longer strides demand. At the same time, individuals with better peripheral muscle function can afford longer and slower strides, because they possess the strength and stability needed to complete gait cycle more efficiently; while those with weak muscle strength may adopt a faster and shorter step mode for biomechanical needs (Kibushi et al., 2019). When both central planning and peripheral muscle strength are impaired, adopting a higher cadence may be a reasonable motor strategy to maintain mobility.

Our interaction term was not statistically significant, and the stratified plots showed nearly parallel lines, suggesting that the central and peripheral factors have an additional effect rather than synergistic effects. This result shows that interventions targeting one mechanism are unlikely to automatically enhance the benefits of interventions targeting another. Optimal cadence improvement requires a combination of intervention for cognitive function and muscle strength at the same time (Gallou-Guyot et al., 2020).

### 4.3 Stride length and turning velocity: unexplained variance and alternative mechanisms

The factors we measured lack significant associations with the stride length and turn velocity, and there are several possible explanations worth discussing. The stride length may be more sensitive to factors that we have not assessed. T2DM peripheral neuropathy can damage the sensory nerves in the feet, resulting in an increase in vibration perception threshold and a decrease in the ability to sense foot position, making the longer steps unstable, independent of cognitive function or overall muscle strength (Dong et al., 2022). In addition, as an alternative indicator of general muscle function, although the grip strength has a good predictive value for mortality and functional decline, it cannot capture the specific impairments of the lower limbs, such as insufficient ankle propulsion or hip extensor weakness, which can directly lead to stride length changes (Escamilla et al., 2025).

Turning is a complex and multi-dimensional task, which may require different neural and muscular resources than walking in a straight line. Turning involves coordinated trunk rotation, asymmetric lower limb movement and dynamic balance adjustments (Zhou et al., 2023). Emerging evidence suggests that turning performance is highly dependent on sensorimotor cortex and vestibular system integrity (Cregg et al., 2020; Aryan et al., 2024). Our MTA score primarily reflects hippocampal atrophy, but turning control may be more sensitive to the function of other brain regions.

The divergent results across gait parameters show that different gait parameters are dominated by distinct control mechanisms. This finding emphasizes the importance of measuring multiple gait dimensions, rather than relying solely on a single composite “gait score.”

### 4.4 Clinical implications: toward brain-centered mobility interventions

These findings are of great significance to clinical practice and intervention development. The current standard management of T2DM mobility protection usually emphasizes glycemic control, neuropathy treatment and strength training prescriptions (Cernea and Raz, 2021). Although these methods remain important, our results suggest that they may not cover the domains with the greatest impact on the most clinically relevant gait outcomes. For gait speed, which is the best parameter for predicting cognitive decline, falls, disability and death, interventions should prioritize brain health maintenance and early dementia risk detection and treatment (Skillbäck et al., 2022). Evidence-based strategies may include intensive early glycemic control to slow down hippocampal atrophy (Canário et al., 2024); cognitive-motor training programs target executive functions, because double-blind randomized controlled trials (RCTs) have shown that these programs can significantly improve walking speed and postural stability (Kao et al., 2018). Aerobic exercise improves both glycemic control and hippocampal neurogenesis, and it is one of the few interventions proven to increase the hippocampal volume in humans (Firth et al., 2018). In addition, aggressive vascular risk factor management, including the control of hypertension and hyperlipidemia, is also conducive to slowing the progression of white matter damage and hippocampal atrophy.

For cadence, its mixed mechanistic characteristics suggest that combined interventions may be the most effective. The optimal strategy should address both domains, adopt dual-task training paradigms that combine cognitive challenges and walking tasks to improve the nervous system’s ability to manage competing demands (Lin et al., 2024); use progressive resistance training to maintain the strength of the lower limb musculature (Khan et al., 2022); and balance and coordination exercises, to challenge the ability of both motor planning and muscular execution (Dunsky, 2019). The negative association between grip strength and cadence suggests that we need to carefully interpret whether “faster cadence” necessarily represents better gait function. In some situations, the increase in step frequency may reflect compensation for weakness rather than functional improvement (Kwon et al., 2017). Clinical evaluation should comprehensively consider the overall gait characteristics, rather than optimizing a certain parameter in isolation.

### 4.5 Study limitations

This study has several limitations. First, due to the cross-sectional design, the causal relationship cannot be established. We cannot conclude that hippocampal atrophy leads to gait slowing. Unmeasured confounders may drive both outcomes; therefore, longitudinal studies are needed to track temporal changes and clarify the causal pathway. Second, we only employed grip strength as an indicator of peripheral muscle function. Grip strength may not fully reflect the specific functional defects of the lower limbs, such as specific muscle strength of the lower limbs, diabetic peripheral neuropathy, body sensory disorders and distal muscle weakness. Future research should include lower limb strength assessment indicators to supplement our research results. Additionally, the full model’s EPV ratio was 5.9:1 given the modest T2DM subgroup (*n* = 41). Bootstrap resampling and a restricted three-predictor model (EPV ≈ 13.7:1) confirmed stable central contributions across cadence (90.4%) and gait speed (54.1% as the largest block, Supplementary Table S1). Future larger cohorts will help refine these effect sizes. Finally, the absence of direct diabetic peripheral neuropathy (DPN) assessment represents a notable limitation: although severe neuropathy was excluded based on clinical examination, we did not quantify neuropathy; sensory neuropathy may independently contribute to gait impairment beyond what is captured by muscle strength, representing a potential source of residual confounding. Our exclusion criteria were extensive, excluding participants with stroke, severe neuropathy, recent falls, major vascular events, and medications affecting gait or cognition. Although these broad exclusion criteria are necessary to isolate specific mechanisms, they may produce healthier and more functionally complete T2DM samples than are usually seen in clinical practice. Therefore, the observed central dominance may be most suitable for relatively stable and walkable elderly T2DM patients, while the real contribution of peripheral factors may be underestimated among elderly people in the real world.

### 4.6 Conclusion

This study revealed a fundamental question about the mechanisms of gait impairment in the older adults with T2DM. The answer depends on the gait parameters examined. For walking speed, the central factors (hippocampal atrophy and cognitive function) account for nearly 90% of explained variance. For cadence, both central and peripheral factors contribute meaningfully, although central factors remain dominant. In contrast, for stride length and turning, neither domain provided substantial explanatory power, suggesting that other mechanisms remain to be explored. These findings challenge the traditional peripheral-focused view of T2DM gait impairment. The preservation of mobility may warrant consideration of brain-centered strategies, such as intensive glycemic control, cognitive training, aerobic exercise and vascular risk management. These strategies are associated with better maintenance of walking speed than approaches focused exclusively on strength training and neuropathy treatment. Although peripheral factors are obviously important for cadence and other gait aspects, brain health should be at the core of any mobility preservation strategy. In the future, these mechanistic insights should be transformed into clinical interventions to test whether slowing hippocampal atrophy and preserving cognitive function can improve walking speed, reduce falls and prolong functional independence.

## Data Availability

The raw data supporting the conclusions of this article will be made available by the authors, without undue reservation.
