# Supplementary material for: Brain over muscle: central mechanisms predominate in gait impairment among older adults with type 2 diabetes mellitus
Source: Front Aging Neurosci. 2026 Jun 17;18:1787033. doi: 10.3389/fnagi.2026.1787033 (PMC13318927; doi:10.3389/fnagi.2026.1787033)
Supplement: Supplementary file 1 [file Table_1.DOCX]

Table S1. Sensitivity Analysis: Hierarchical Regression Results

| **Outcome** | **Model** | **Block** | **deltaR2** | **Boot_CI** | **p_value** | **Percent_model_R2** | **Total_model_R2** |
| --- | --- | --- | --- | --- | --- | --- | --- |
| Gait speed (cm/s) | Full model (EPV ~ 5.9:1) | Central (MTA + MoCA) | 0.181 | - | 0.007 | 87.1% | 0.489 |
|  |  | Peripheral (Grip strength) | 0.027 | - | 0.197 | 12.9% | 0.489 |
|  | Restricted model (EPV ~ 13.7:1) | Central (MTA + MoCA) | 0.129 | 0.020-0.404 | 0.055 | 54.1% | 0.239 |
| Cadence (steps/min) | Full model (EPV ~ 5.9:1) | Central (MTA + MoCA) | 0.269 | 0.089-0.462+ | 0.003 | 60.1% | 0.500 |
|  |  | Peripheral (Grip strength) | 0.179 | 0.053-0.318+ | 0.002 | 39.9% | 0.500 |
|  | Restricted model (EPV ~ 13.7:1) | Central (MTA + MoCA) | 0.248 | 0.082-0.497 | 0.004 | 90.4% | 0.275 |
| Stride length (cm) | Full model (EPV ~ 5.9:1) | Central (MTA + MoCA) | 0.037 | - | 0.391 | 64.6% | 0.374 |
|  |  | Peripheral (Grip strength) | 0.020 | - | 0.310 | 35.4% | 0.374 |
|  | Restricted model (EPV ~ 13.7:1) | Central (MTA + MoCA) | 0.032 | 0.003-0.209 | 0.474 | 13.4% | 0.235 |
| Turn velocity (deg/s) | Full model (EPV ~ 5.9:1) | Central (MTA + MoCA) | 0.095 | - | 0.068 | 70.3% | 0.486 |
|  |  | Peripheral (Grip strength) | 0.040 | - | 0.118 | 29.7% | 0.486 |
|  | Restricted model (EPV ~ 13.7:1) | Central (MTA + MoCA) | 0.125 | 0.026-0.352 | 0.043 | 38.6% | 0.323 |
| Note. EPV, events-per-variable; MTA, medial temporal atrophy; MoCA, Montreal Cognitive Assessment. deltaR2 represents the incremental variance explained by each predictor block. Percent of model R2 refers to the proportion of the total model R2 attributable to that block. Bootstrap 95% CIs were derived from 1,000 resamples. + Values reported in the primary analysis (Results section). - Not applicable. | | | | | | | |
